# Digital Transformation of Grocery In-Store Shopping-Scanners, Artificial Intelligence, Augmented Reality and Beyond: A Review

**DOI:** 10.3390/foods13182948

**Published:** 2024-09-18

**Authors:** Radosław Wolniak, Kinga Stecuła, Barış Aydın

**Affiliations:** 1 Department of Economics and Informatics, Faculty of Organization and Management, Silesian University of Technology, 44-100 Gliwice, Poland; 2 Department of Production Engineering, Faculty of Organization and Management, Silesian University of Technology, 44-100 Gliwice, Poland; 3 Department of Industrial Engineering, Faculty of Engineering and Natural Sciences, Manisa Celal Bayar University, Manisa 45140, Türkiye; baariisaydin35@gmail.com

**Keywords:** grocery, food, shopping, scanners, AI, shopping applications, AR, facial recognition

## Abstract

This paper reviews the digital transformation of grocery shopping, focusing on the technological innovations that have redefined consumer experiences over the past decades. By analyzing both academic literature and up-to-date information from websites, the study provides a review of the evolution of grocery shopping from traditional methods to modern, technology-driven approaches. The review categorizes developments into two primary areas: in-store and online grocery shopping. In-store shopping has progressed from traditional interactions to the implementation of self-service checkouts, handheld scanners, mobile apps, and AI-based solutions, including augmented reality (AR) and facial recognition. This paper reviews the first area which are in-store technological solutions. This study aims to highlight the revolution in grocery shopping from a technological perspective, present the most significant digital achievements, and outline the future possibilities for further advancements in this field.

## 1. Introduction

The rapid advancement of technology has led to significant transformations in various aspects of daily life. It also refers to the way of shopping, including grocery shopping. As consumers increasingly seek convenience, efficiency, and personalized experiences, both in-store and online grocery shopping have evolved in response to these demands. This paper reviews the digital transformation in grocery in-store shopping, exploring the technological innovations that have redefined the shopping experience over the decades. In recent years, the landscape of grocery shopping has been reshaped by the introduction of various digital tools and solutions. From the implementation of self-service checkouts and handheld scanners in physical stores to the emergence of AI-driven solutions and augmented reality (AR) applications, technology has revolutionized the way consumers interact with grocery stores.

Shopping has always been a fundamental part of human society, evolving from simple barter systems to complex retail systems. Over the centuries, the way people shop has undergone numerous revolutions, driven by changes in technology, the economy, and consumer behavior. The Industrial Revolution marked a significant shift, introducing mass production and department stores, while the advent of the Internet gave rise to e-commerce, forever altering the shopping landscape. Today, we are witnessing another revolution in shopping, fueled by digital innovations that blend the physical and virtual worlds, offering consumers unprecedented convenience, personalization, and immersive experiences. This ongoing transformation reflects the continuous adaptation of shopping practices to meet the evolving needs and expectations of modern consumers. It should also be added that, on the one hand, consumers expect new ways of shopping, making payments, and receiving parcels. On the other hand, manufacturers and service providers also compete with each other in the battle for customers.

In recent years, the digital transformation of grocery shopping has garnered significant academic attention. This transformation can be conceptualized through the lens of technological determinism, which posits that technological advances are the primary drivers of societal and behavioral change [1]. The shift from manual, in-person shopping to digitally augmented experiences underscores how modern grocery shopping is becoming more aligned with contemporary consumers’ expectations of speed, accuracy, and personalized service [2]. Digital tools, including self-service checkouts, AI-powered applications, augmented reality (AR) systems, and biometric recognition technologies, have not only redefined in-store consumer experiences, but have also led to changes in retail business models [3,4].

From a socio-technical perspective, this transformation can be further understood as a response to the growing intersection of human agency and technological systems [5]. Modern consumers no longer interact with products; they engage in dynamic, technology-mediated environments that combine physical and virtual worlds [6]. This paradigm shift in consumer behavior is reflected in the rapid acceptance of self-service technologies (SST) and solutions driven by artificial intelligence (AI), which offer increased convenience and improve operational efficiency for retailers [7].

Digital transformations in grocery shopping have introduced a reconfiguration of consumer roles, in which consumers now actively participate in co-creating their shopping experiences [8]. Integration of AI technologies, for example, allows personalization of in-store experiences by offering customized product suggestions and interactive recommendations based on previous shopping behavior and real-time preferences [9].

The competitive pressures faced by retailers have led to the development of innovative solutions that not only meet but anticipate consumer needs [3]. Grocery stores now employ advanced systems such as real-time inventory management, dynamic pricing algorithms, and seamless checkout processes, which are designed to optimize both consumer satisfaction and operational efficiency [4,8]. This competitive innovation can be contextualized within the broader framework of market-driven technological advancements, which emphasize the interaction between technological affordances and consumer demand [9].

Taking into account the dynamic nature of this field, the authors have conducted a review of both academic literature and current information available on websites. The paper presents and discusses the most important technological achievements, innovative solutions, and future directions (expectations) in the field of grocery in-store shopping. Starting from traditional shopping, the paper refers to self-service checkouts, scanners, mobile applications for grocery shopping, and different AI-based solutions applied in-stores, including facial recognition and augmented reality usage.

This paper seeks to systematically explore the technological revolution in grocery in-store shopping, providing a comprehensive review of how digital tools—such as self-service checkouts, AI, AR, and facial recognition—have redefined traditional shopping experiences. By analyzing both the evolution of these innovations and their current applications, this study aims to present a detailed understanding of the intersection between technological advancements and consumer behavior, as well as to forecast future developments that could further transform the grocery retail landscape.

## 2. Materials and Methods

Review refers not only to the literature but also to websites, as the topic is centered on current and future solutions related to grocery in-store shopping. Innovative and future solutions concerning grocery shopping are often presented by specific stores and supermarkets on their websites, and literature hardly presents such up-to-date trends. Therefore, the authors analyzed the literature on grocery shopping technology and its change over decades, but also made review research searching the Internet. The extension of the review beyond the literature is justified by the fact that many innovative trends are often first introduced by retailers in practice/online before being discussed in scientific publications. Then, relying on web sources is necessary due to the emerging nature of the topic. Another reason for using Internet sources is that this paper concerns the latest industry trends that have not been studied in the academic field yet. Moreover, for many of the latest and future technologies that are described and discussed in this paper, there is limited or no academic research.

As a results of screening papers and reviewing websites, the authors divided the topic of grocery shopping into two main categories, each reflecting the distinct yet interconnected pathways of technological development in this domain:

- in-store grocery shopping;
- on-line grocery shopping.

**Figure 1:**
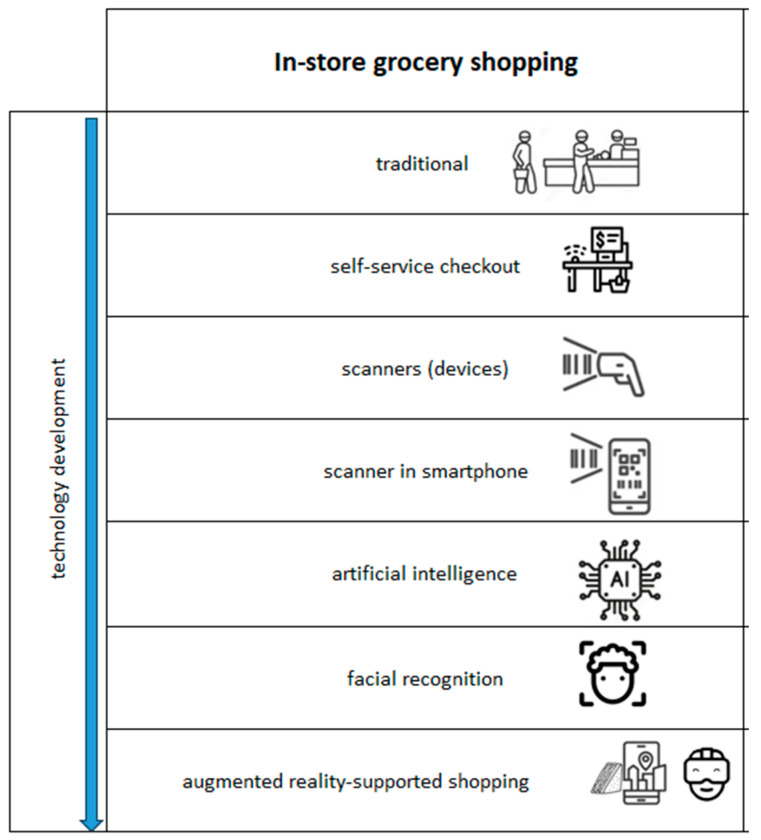
Scheme of the most important points during the digital transformation of grocery in-store shopping in the context of technological development.

In this paper, the authors present the evolution of grocery in-store shopping from a technological perspective, highlighting various stages of technology development. Traditional in-store shopping has undergone significant changes, beginning with self-service checkouts, followed by the introduction of handheld scanners and mobile apps for scanning items in supermarkets. The paper further explores AI-based solutions that enhance the shopping experience, culminating in the integration of augmented reality to assist shoppers. Figure 1 shows the scheme of the most important points during the digital transformation of grocery in-store shopping in the context of technological development. According to this key, the authors describe the subsequent sections in the paper—this paper presents the first part of the review as it shows in-store shopping.

The objectives of this review include the following:

- O1: Showing the revolution in grocery in-store shopping from a technological perspective;
- O2: Gathering important technological achievements in grocery in-store shopping in one paper
- O3: Presenting the latest technological achievements available in stores in the field of grocery shopping.
- O4: Exploring the potential future directions of grocery in-store shopping.

## 3. In-Store Grocery Shopping: Review Results

### 3.1. From Traditional Shopping to Self-Service

In recent years, the landscape of grocery shopping has undergone a significant transformation, driven mainly by advancements in technology. The traditional way of grocery shopping, characterized by physical visits to supermarkets or local stores, involved manually selecting items from shelves, queuing at checkout counters, and interacting with store personnel. This conventional approach served as the foundation of retail shopping for decades, focusing on customer service and in-person interactions.

The advent of self-service checkout terminals marked an important moment in the evolution of grocery shopping. These terminals allow customers to scan and bag their own items, providing autonomy and reducing checkout times. As Collier and Kimes [10] notice, self-service technologies (SSTs) helps to reduce labor costs while providing more channel options. By eliminating the need for direct cashier assistance, self-service checkout can enhance efficiency and convenience, catering to the preferences of time-conscious shoppers. On the other hand, customers must be convinced to use these checkouts. The results of the research presented in [11] shows that there are eight features connected with performance that are important for customers to choose self-service checkout instead of traditional checkout. They include the following:

- usefulness,
- speed,
- efficiency,
- consistency,
- cost-effectiveness,
- user-friendliness,
- reliability,
- trialability

Moreover, there are three features related with convenience which are:

- locational convenience,
- time convenience,
- physical exertion.

It must be also noticed that using these checkouts can be particularly appreciated during busy periods or when only a few items are being purchased. Moreover, self-service checkout can be faster than traditional checkout lanes, especially for shoppers who are familiar with the process and proficient at scanning items.

However, self-service checkout can not be perceived as flawless. Actually, there are many issues and sometimes customers refuse to use them. Some people may find the technology challenging to use, leading to frustration and delays. This is especially true for older adults or those who are not accustomed to technology. As Dean [12] concludes based on his research, compared to younger consumers, older consumers more rare experience SSTs and they are less confidence in using SSTs. Elderly also reported missing human interaction a lot. In general, they also decided used self-checkout less than younger customers. Pentzold and Bischof [13] in their paper make a reflection that self-checkout systems, despite being designed for individual use, often require collective effort to operate effectively due to their susceptibility to human error and technical malfunctions, highlighting a complex interaction between human and machine performance. What is more, the frequency of service failures significantly influences customers’ attitudes towards self-service technology; while initial problems may be tolerated, repeated issues lead to a preference for traditional checkouts, and efficient, effective recovery efforts are essential to maintain customer satisfaction and positive perceptions of SST [14]. As Lyu et al. [15] sums up, improving service quality is crucial for increasing customer acceptance and satisfaction in self-service retail stores, boosting the likelihood of customers revisiting and providing positive word-of-mouth feedback. Enhancing the shopping experience and leveraging new technologies can attract more customers to self-service retail stores, increasing both customer convenience and corporate profitability.

**Figure 2:**
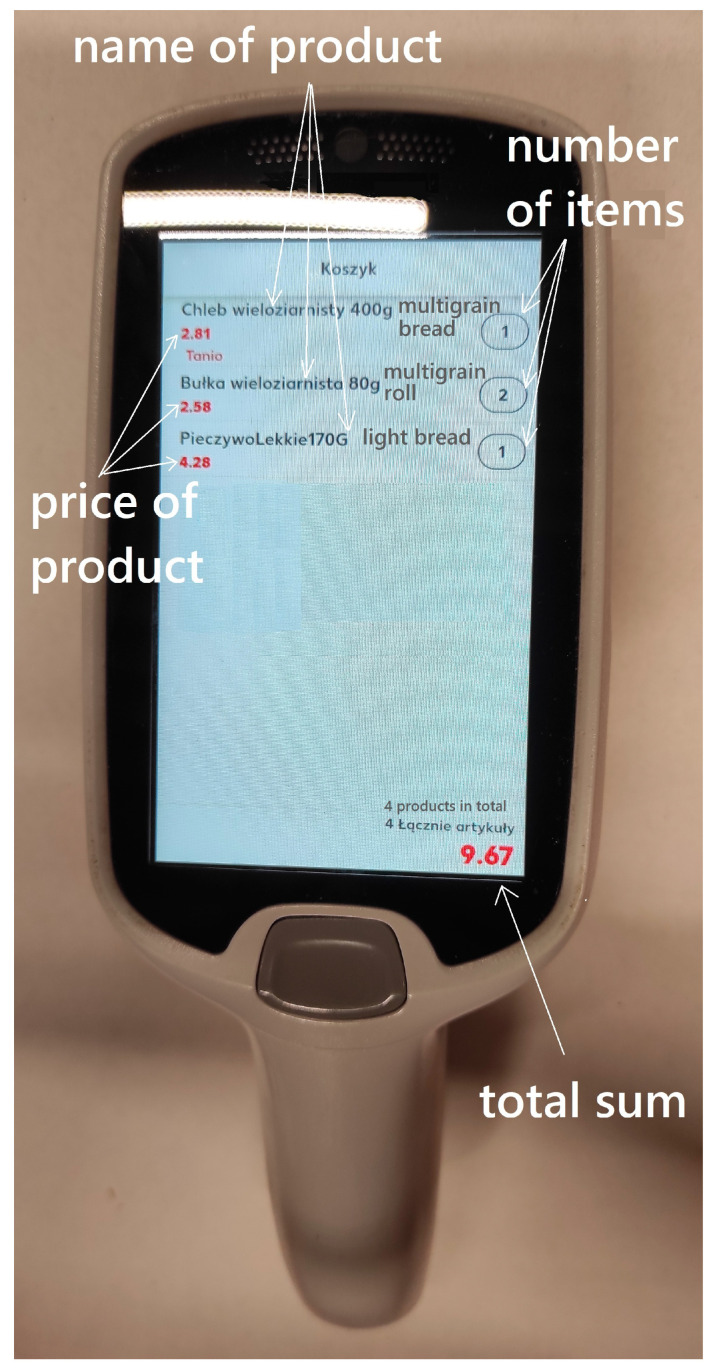
The general view of the scanner and its digital elements [own work].

**Figure 3:**
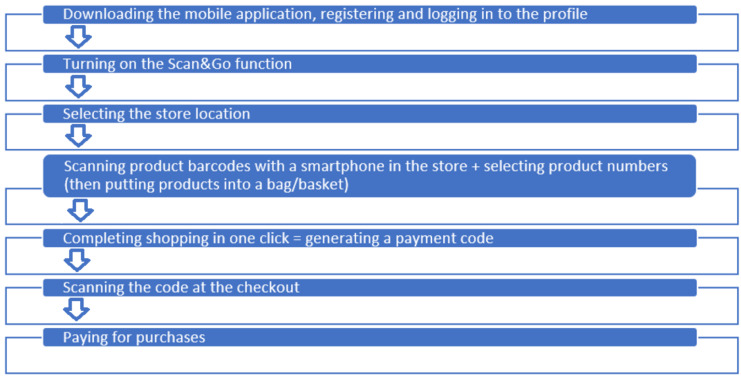
The scheme of Scan&Go technology using a smartphone [own work].

Scan&Go technology represents a further innovation in self-service shopping. Customers utilize handheld scanners (store-provided devices) or their own smartphones to scan products as they shop, placing items directly into their bags. This technology can have different names depending on the shop. When it comes to a special scanner (Figure 2), this device can be taken by a client free of charge in the store of a given supermarket. Then, the products can be scanned and this way the customer creates their own shopping list. Moreover, products can also be removed from the device if the customer decides not to purchase a given product. Customers can also use their own smartphones, but to do so they must download a special application related to a given store or supermarket in the first place. This method provides a seamless and personalized shopping experience, enabling shoppers to track spending in real-time and access digital coupons or promotions. Figure 3 shows the scheme of Scan&Go technology using a smartphone.

Currently, more and more supermarkets or shops offer such a possibility. Examples of supermarkets around the world are presented in Table 1. Table 1 contains only Internet sources due to the fact that Scan&Go technology is an emerging trend, has not been studied in the academic field, and the aim of this paper is to present current and up-to-date data. This approach eliminates the traditional checkout process. Upon completion, shoppers finalize their purchases at a designated terminal, making payment quickly and leaving the store without delay.

There are plenty of benefits connected with this technology. The most important include:

- Customers can scan items as they shop, eliminates the need to place products on a belt conveyor, scan them and then pack them into bags, and also reducing or eliminating the need to wait in checkout lines.
- Ability to add or remove items from the virtual cart at any time.
- Control of purchases by the customer in terms of products, their prices and the overall total payment for shopping.
- Instant price and product information helps customers make informed purchasing decisions.
- Ability to track spending in real-time and manage budgets more effectively.
- Fewer staff required at checkout counters, allowing resources to be allocated to other areas of the store.
- Automated systems ensure accurate pricing and inventory management.
- Faster, contactless payment options through the app or dedicated terminals.
- Mobile apps provide additional features such as digital receipts and shopping lists.
- Reduced congestion at traditional checkout areas.

Despite numerous advantages, the solution of scanning products with a scanner or smartphone may involve certain problems and threats. Malfunctions or glitches in scanners or apps can disrupt the shopping process and cause frustration. Connectivity issues with smartphones can hinder the use of mobile scanning apps.

Another great problem is the increased risk of theft or misuse, such as under-scanning items or intentional scanning errors. Customers may make mistakes in scanning items, leading to incorrect totals or missed products. Moreover, the absence of direct supervision and reduced staff presence in Scan and Go areas can create opportunities for theft. Customers may intentionally under-scan or fail to scan items, exploiting the lack of immediate oversight. Some individuals may find and exploit vulnerabilities in the scanning system, such as barcodes that do not register correctly. On the other hand, shops rely on security cameras and periodic checks, however, they may not be sufficient to deter or catch all instances of theft. Then, increased theft can lead to higher operational costs due to loss of inventory. On the other hand, legitimate customers may feel uncomfortable or distrusted due to increased security measures, affecting their shopping experience. There is also an issue connected with the potential for false accusations or disputes if customers are mistakenly suspected of theft.

While Scan&Go technology enhances convenience and efficiency, it also introduces challenges related to theft and security that must be carefully managed to protect store assets and ensure a fair shopping environment for all customers.

### 3.2. Artificial Intelligence 

AI technology can be used in various aspects of grocery shopping. Artificial intelligence has increasingly become a transformative force in the buying process of in-store grocery shopping, primarily due to its ability to enhance efficiency, improve customer experiences, and optimize inventory management [25]. The integration of AI technologies into the grocery shopping experience is driven by the need for retailers to meet the evolving demands of consumers while maintaining competitive advantage in a rapidly changing market. In the Table 2 there are examples of AI usage in grocery in-store shopping [9,26].

Smart shopping assistants have emerged as a valuable tool in grocery stores, significantly enhancing the shopping experience for customers [47,51]. These AI-powered systems can be found in various forms, including mobile applications, in-store kiosks, and voice-activated devices [27]. By providing real-time assistance, smart shopping assistants help customers navigate stores more efficiently, find products, and make informed purchasing decisions [28,29,30,31,32,33,34].

The example of a smart shopping assistant is the Amazon Alexa integration used in select grocery stores [57]. Customers can use voice commands to ask Alexa about product locations, receive cooking suggestions, or add items to their shopping lists. This hands-free interaction allows shoppers to easily find ingredients for recipes or locate specific products in-store without having to search the aisles manually. As a result, the shopping experience becomes more convenient and engaging, encouraging customers to explore new products and make additional purchases [58]. Another example is the Walmart app, which incorporates a shopping assistant feature that helps customers create shopping lists based on their preferences and previous purchases [59]. The app uses location-based services to guide customers through the store, displaying the quickest routes to find items on their lists. By streamlining the shopping process, Walmart’s app minimizes the time spent wandering the aisles and enhances overall customer satisfaction [60].

In some grocery stores, such as Kroger, smart shopping assistants are implemented through in-store kiosks equipped with touchscreen interfaces [61]. Customers can use these kiosks to access product information, check prices, and find current promotions. Additionally, the kiosks can provide personalized recommendations based on customer profiles, helping shoppers discover new items they may not have considered otherwise. This interactive experience not only saves time but also enriches the shopping journey by making it more informative and enjoyable [62]. Also, Albertsons has introduced a mobile app that features a shopping assistant that allows customers to scan items in-store to check prices and nutritional information [63]. This feature empowers consumers to make healthier choices and compare products before purchasing. The app also provides personalized deals and suggestions based on past shopping behavior, fostering customer loyalty and enhancing the overall experience [64].

In the Table 3 there is an juxtaposition of using of smart shopping in the case of in-store grocery shopping.

Dynamic pricing strategies have emerged as a vital tool in the area of in-store grocery shopping, allowing retailers to adjust prices in real-time based on various factors such as demand fluctuations, competitor pricing, and customer behavior. This approach is increasingly being adopted by grocery stores worldwide, to enhance profitability and optimize sales [39].

Dynamic pricing technology in grocery stores represents a sophisticated approach to pricing that leverages real-time data analysis and algorithms to adjust prices based on various factors [49]. This technology, which has gained traction in recent years, enables retailers to optimize their pricing strategies in response to fluctuating market conditions, customer behaviors, inventory levels, and competitive pressures [41].

Dynamic pricing relies on the integration of advanced data analytics and machine learning algorithms. These systems collect and analyze vast amounts of data from multiple sources, including sales history, customer traffic patterns, weather forecasts, and even social media trends. By synthesizing this information, grocery retailers can gain insights into consumer demand and preferences, allowing them to make informed decisions about price adjustments [42,43].

One of the primary advantages of dynamic pricing technology is its ability to respond quickly to changes in market conditions. For instance, if a grocery store observes an increase in demand for a particular product, perhaps due to a seasonal trend or a local event, the system can automatically raise prices to maximize revenue [44]. Conversely, if inventory levels are high for a specific item and sales are sluggish, the technology can lower prices to stimulate demand and reduce excess stock. This level of responsiveness is particularly crucial in the grocery sector, where perishable goods necessitate careful management to minimize waste and maximize profitability [45]. Also, dynamic pricing technology also enables retailers to implement personalized pricing strategies. By analyzing individual customer data, such as shopping habits and preferences, grocery stores can tailor prices to specific segments of their customer base. For instance, loyalty program members might receive exclusive discounts on items they frequently purchase, creating a more personalized shopping experience that encourages repeat business and enhances customer loyalty [46].

Despite its many advantages, the implementation of dynamic pricing technology in grocery stores is not without challenges. One significant concern is the potential for customer backlash if price fluctuations are perceived as unfair or manipulative. Customers may feel frustrated if they notice that prices for the same item vary significantly from one visit to another. To mitigate this risk, retailers must strike a delicate balance between optimizing pricing for profitability and maintaining customer trust and satisfaction [48,49].

In recent years, the rise of big data and the Internet of Things (IoT) has further augmented the capabilities of dynamic pricing technology. By harnessing data from various sources, including social media, economic indicators, and real-time sales data, businesses can achieve an unprecedented level of insight into market conditions and consumer preferences. As a result, dynamic pricing is becoming more accurate and responsive, allowing companies to implement pricing strategies that are not only reactive but also proactive [40,50].

In Poland, a notable example is Carrefour, which has implemented dynamic pricing through its digital price tags. These electronic tags are connected to a central system that allows for instantaneous updates to pricing information [73]. By using these digital price tags, Carrefour can quickly modify prices based on various factors, such as product demand, competitor pricing, or the proximity of expiration dates for perishable goods. In addition to digital price tags, Carrefour utilizes data analytics and artificial intelligence to inform its pricing decisions. The retailer collects vast amounts of data from customer purchases, foot traffic, and market trends. By analyzing this data, Carrefour can gain insights into customer behavior and preferences, allowing the company to make informed pricing adjustments that align with consumer demand. This capability enables Carrefour to implement targeted promotions and discounts, enhancing customer satisfaction while minimizing waste [74].

Also, Carrefour’s dynamic pricing technology integrates seamlessly with its inventory management systems. This integration ensures that price changes reflect current stock levels and product availability, allowing the retailer to optimize inventory turnover. For example, when stock levels for a specific product decrease, Carrefour can lower the price to encourage quicker sales, or conversely, increase prices for high-demand items.

The combination of digital price tags, data analytics, and seamless integration with inventory management systems empowers Carrefour to implement dynamic pricing strategy. This technology not only enhances operational efficiency but also improves the shopping experience for customers by offering timely and relevant pricing adjustments.

Another Polish retailer, Zabka, has begun experimenting with dynamic pricing in select locations. By analyzing foot traffic data and sales trends, Zabka can adjust prices on popular items throughout the day. For example, during peak shopping hours, prices on everyday essentials might remain stable, while off-peak hours could see discounts to drive traffic. This approach allows Zabka to optimize sales based on customer purchasing patterns, ultimately enhancing overall profitability [75].

In other countries, Walmart has been a pioneer in employing dynamic pricing strategies, utilizing advanced algorithms to monitor competitor prices and adjust its own pricing accordingly. This enables Walmart to remain competitive while maximizing profit margins. For example, if a competitor lowers the price of a specific cereal brand, Walmart’s system can automatically adjust its pricing to match or beat the competitor’s offer, ensuring that customers perceive Walmart as the most cost-effective option [76]. Similarly, Tesco in the United Kingdom has integrated dynamic pricing into its business model through its Clubcard loyalty program. The retailer analyzes customer purchase history and market trends to tailor discounts and special offers to individual shoppers. For instance, if a customer frequently purchases organic products, Tesco might provide targeted discounts on organic items, encouraging repeat purchases while maximizing customer loyalty [77].

Dynamic pricing strategies also play an important role in managing promotional events and seasonal sales. For instance, during holiday seasons, grocery stores can implement temporary price reductions on festive products, attracting customers who may be looking to stock up. By analyzing real-time sales data, retailers can adjust these promotional prices to ensure they remain competitive and appealing to customers.

In the Table 4 there is an juxtaposition of using of dynamic pricing strategy in the case of in-store grocery shopping.

AiFi is a technology that enables the development of fully automated grocery stores, utilizing artificial intelligence and advanced sensor systems to provide a seamless shopping experience without the need for traditional checkout processes [1]. It is connected with usage of RFID technology. RFID (Radio-Frequency Identification) technology is increasingly being utilized in grocery stores to enhance operational efficiency, improve inventory management, and elevate the customer shopping experience. This technology relies on small, electronic tags embedded with microchips that store data, which can be transmitted via radio waves to RFID readers. These readers capture the information and send it to a central database, where it can be analyzed and used in various applications [82,83].

One of the important uses of RFID technology in grocery stores is in inventory management. Unlike traditional barcodes, RFID tags do not require a direct line of sight to be scanned. This allows for faster and more accurate tracking of products throughout the store. As goods are received, the RFID tags on each item or pallet can be scanned automatically, updating the store’s inventory system in real-time [84]. This enables store managers to maintain a more accurate count of stock levels, reducing the likelihood of out-of-stock situations and minimizing the risk of overstocking. It also simplifies the process of conducting inventory audits, as large quantities of items can be scanned simultaneously without the need to handle each item individually [85].

In terms of customer experience, RFID technology can significantly streamline the checkout process. In an RFID-enabled store, customers could potentially place all their items in a cart and simply walk through an RFID scanner, which would instantly read the tags on all items and generate a total bill [86]. This eliminates the need for scanning each item individually at the checkout counter, thereby reducing wait times and improving the overall shopping experience. While this full-scale implementation is still in its early stages, some stores are experimenting with hybrid systems that combine RFID and traditional barcoding to speed up the checkout process [87].

This innovative approach allows customers to shop for groceries, place items in their carts, and leave the store without having to go through a conventional checkout line, as the system automatically tracks purchases and calculates the total cost of their shopping cart in real-time [88,89]. In an AiFi-enabled store, customers typically enter through a turnstile that scans their mobile app or identification, granting them access to the shopping area. As they navigate the aisles, a combination of cameras, sensors, and AI algorithms monitors their selections, automatically adding items to their virtual shopping carts. This technology relies on computer vision to accurately identify products and track their movement, ensuring that every item is accounted for [90].

Once customers have completed their shopping, they can simply exit the store, and the system processes the payment automatically through their registered accounts. The total cost is calculated based on the items detected in their virtual cart, providing a quick and efficient way to complete transactions without any manual scanning or waiting in line. This eliminates the hassle of traditional checkout processes and significantly reduces the time spent in the store [91,92].

In Poland, the concept of automated stores utilizing AiFi technology is gaining traction, with several retailers exploring or implementing this innovative solution. For instance, Zabka, a popular convenience store chain, has begun testing automated formats that incorporate advanced technology to enhance the shopping experience [93]. Their stores aim to provide customers with a seamless, cashier-less experience that aligns with the growing demand for convenience and efficiency in grocery shopping. The integration of AiFi technology in automated stores not only benefits customers through a streamlined shopping experience but also helps retailers optimize their operations. By reducing the need for staff at checkout stations, grocery stores can reallocate resources to improve customer service in other areas. Additionally, the data collected through these automated systems can offer valuable insights into shopping habits and preferences, allowing retailers to tailor their offerings and marketing strategies effectively [94].

In the Table 5 there is an juxtaposition of using of AiFi technology in the case of in-store grocery shopping.

The benefits of using AI technology in in-store grocery shopping are profound and transformative, enhancing both the customer experience and operational efficiency for retailers (Table 6). One of the most significant advantages is the ability to provide a personalized shopping experience. By analyzing customer data, AI algorithms can recommend products tailored to individual preferences, track buying habits, and send targeted promotions that resonate with specific shopper demographics. This level of personalization fosters customer loyalty and increases the likelihood of repeat visits, ultimately driving sales [9,25,26,107].

Another key benefit is improved inventory management. AI technology can predict demand patterns by analyzing historical sales data, seasonal trends, and external factors, enabling retailers to optimize stock levels. This proactive approach helps to reduce instances of overstocking or stockouts, ensuring that popular products are always available for customers. As a result, grocery stores can minimize waste associated with expired products, enhancing overall operational efficiency and sustainability. AI also streamlines the checkout process, reducing wait times for customers. With the implementation of automated checkout systems, such as self-service kiosks and mobile scanning applications, shoppers can complete transactions quickly and efficiently. This convenience not only enhances the customer experience but also allows staff to focus on other important tasks within the store [29,37,38,44].

AI technology facilitates dynamic pricing strategies, enabling grocery retailers to adjust prices based on market trends, competitor pricing, and customer demand in real-time. This flexibility allows stores to optimize their pricing strategies, attract price-sensitive customers, and maximize profit margins [46,48,49,50]. Another significant benefit of AI in grocery shopping is its capability to enhance security and fraud detection. AI systems can analyze surveillance footage and monitor customer behavior, identifying suspicious activities and potential theft. This added layer of security contributes to a safer shopping environment for both customers and employees [9,25,26,29].

The integration of AI technology in in-store grocery shopping presents several challenges that retailers must address to maximize its potential benefits (Table 7). One of the most pressing issues is data privacy concerns. As AI systems often rely on extensive data collection to function effectively, customers may feel apprehensive about sharing their personal information. The fear of data breaches and the potential misuse of their data can lead to resistance among consumers. To alleviate these concerns, grocery retailers need to implement clear and transparent data privacy policies, ensuring customers understand how their information is used and protected [9,26,51,111].

Another significant challenge is the integration of AI technology with existing systems and processes. Many grocery retailers operate with legacy systems that may not seamlessly accommodate new AI solutions. This incompatibility can lead to operational disruptions, increased costs, and a steep learning curve for staff who need to adapt to new technology [111,112]. To overcome this hurdle, retailers should conduct thorough assessments of their current infrastructure and develop a strategic plan for integrating AI in a phased and manageable manner.

High implementation costs also pose a considerable barrier to the widespread adoption of AI technology in grocery stores. The initial investment in AI software, hardware, and training can be substantial, deterring some retailers from pursuing these innovations [111]. To mitigate this challenge, grocery retailers can consider starting with pilot programs that demonstrate the return on investment before committing to broader implementations. Seeking partnerships with technology providers or exploring government grants may also provide financial support to offset the costs. Also, there is often resistance from staff when new technologies are introduced. Employees may fear job displacement or feel overwhelmed by the need to learn new skills. To foster a positive transition, grocery retailers should prioritize employee training and emphasize the collaborative benefits of AI, positioning it as a tool to enhance their roles rather than replace them.

Bias in AI algorithms presents another challenge that retailers must address. If the training data used to develop AI systems is biased, the resulting recommendations and decisions can also be biased, potentially leading to unfair treatment of certain customer groups [113,114,115]. Regular audits and diverse datasets can help mitigate this risk, ensuring that AI applications remain fair and equitable. It can be stated, that the reliability of AI systems is a critical consideration. Technical failures or inaccuracies can disrupt operations and lead to customer dissatisfaction. Retailers need to implement robust testing and maintenance protocols to ensure the reliability of AI technologies. Additionally, having backup systems in place can help manage any disruptions that may arise [42,115].

**Table 7:** Challenges of using AI technology-in-store grocery shopping.

Challenge	Description	Methods of Overcoming
Data Privacy Concerns	The implementation of AI technology often requires the collection of customer data, raising concerns about privacy and data security. Customers may be hesitant to share personal information.	Grocery stores can establish clear data privacy policies, ensuring transparency about data usage. Additionally, they can implement strong data security measures to protect customer information.
Integration with Existing Systems	Integrating AI technology with legacy systems and processes can be complex and costly. Compatibility issues may arise, leading to operational disruptions.	Grocery stores can conduct thorough assessments of their existing systems before implementing AI. Collaborating with technology partners can help ensure smooth integration and minimize disruptions.
High Implementation Costs	The initial costs associated with adopting AI technology can be significant, including expenses for software, hardware, and training staff.	Grocery stores can consider phased implementation, starting with pilot programs to demonstrate ROI. Additionally, seeking government grants or partnerships with tech firms can help offset costs.
Staff Resistance to Change	Employees may resist the introduction of AI technology due to fears of job loss or the need to adapt to new systems and processes.	Providing training and support for staff is essential. Grocery stores should communicate the benefits of AI in enhancing their roles rather than replacing them, fostering a culture of collaboration.
Complexity of AI Algorithms	The algorithms used in AI can be complex and require specialized knowledge to develop and maintain. This complexity can lead to challenges in understanding and interpreting AI outputs.	Grocery stores can invest in training staff or hiring data scientists and AI specialists to ensure a thorough understanding of AI technology. Utilizing user-friendly AI solutions can also simplify usage.
Bias in AI Systems	AI algorithms can unintentionally reflect biases present in training data, leading to unfair treatment of certain customer groups or inaccurate recommendations.	Regular audits of AI systems should be conducted to identify and mitigate bias. Diverse datasets should be used for training algorithms, and ongoing monitoring can help ensure fairness and accuracy.
Reliability of Technology	AI systems may experience downtime or inaccuracies, leading to disruptions in service and customer dissatisfaction.	Implementing robust testing and maintenance schedules can enhance system reliability. Having backup systems and manual processes in place can help mitigate the impact of technology failures.
Regulatory Compliance	Compliance with data protection regulations (such as GDPR) and other legal frameworks can pose challenges for grocery stores using AI technology.	Grocery stores should consult legal experts to ensure compliance with all relevant regulations. Developing a compliance framework that incorporates data management and privacy policies is crucial.

Source: Author’s own work on basis [9,25,26,29,30,32,33,34,35,36,37,38,43,44,45,46,47,48,49,50,51,53,54,55,89,90,91,92,107,112,115,116].

### 3.3. Facial Recognition Technology

Facial recognition technology is increasingly being integrated into grocery shopping, offering a novel and efficient way for customers to complete their purchases [116]. This technology, often referred to as “FacePay”, allows customers to make payments using their facial biometrics, eliminating the need for traditional payment methods such as cash, credit cards, or even mobile devices. As this technology gains traction globally, it is poised to transform the grocery shopping experience by enhancing convenience, speed, and security [117,118,119].

In practice, facial recognition payment systems work by linking a customer’s facial data to their payment information. During the checkout process, a camera scans the customer’s face, and the system automatically verifies their identity and processes the payment [120]. This method of payment is particularly advantageous in environments where speed and efficiency are critical, such as grocery stores, where long checkout lines can be a significant pain point for customers [121].

The usage of Facepay at Empik involves a seamless integration of advanced technology into the payment process. Customers who wish to use this service first need to register their facial data in the Empik system. This registration can typically be completed through the Empik app or at designated kiosks within the store. Once registered, the customer’s facial features are securely stored, allowing for quick and efficient identification during future transactions. When a customer decides to make a purchase using Facepay, they simply approach the checkout area where the Facepay technology is enabled. The system uses high-resolution cameras equipped with facial recognition software to scan the customer’s face. Upon successful identification, the system retrieves the customer’s linked payment information, and the transaction is processed automatically without the need for physical payment methods such as cards or cash [122].

This innovative payment method offers several benefits to both customers and the retailer. For customers, Facepay provides a fast and convenient checkout experience, eliminating the need to fumble for wallets or payment cards. It enhances security as payments are processed through biometric verification, reducing the risk of fraud associated with traditional payment methods. Additionally, the technology aligns with the growing trend of contactless transactions, which has become increasingly important in the context of health and safety. For Empik, implementing Facepay not only enhances customer satisfaction but also helps streamline the checkout process, reducing wait times and improving overall efficiency in the store. By adopting such cutting-edge technology, Empik positions itself as a forward-thinking retailer, catering to the preferences of modern consumers who value convenience and speed in their shopping experiences. This initiative reflects Empik’s commitment to leveraging technology to enhance customer engagement and drive sales in an increasingly competitive retail landscape.

Several other grocery chains have adopted facial recognition technology. Hema Supermarkets, operated by Alibaba in China, have integrated FacePay with the Alipay system, offering customers a high-tech shopping experience that includes automated checkouts and personalized recommendations [123]. Similarly, Metro AG in Germany and Auchan Retail in several European countries have begun experimenting with facial recognition payments, aiming to reduce checkout times and improve overall customer satisfaction [124].

The benefits of facial recognition payments in grocery shopping are clear: they offer unparalleled convenience, reduce wait times, and provide a hygienic, contactless payment option. It should be mentioned, that the implementation of this technology is not without challenges [117,118,125]. Concerns about privacy and data security are significant, as facial recognition involves the collection and storage of sensitive biometric data. To address these concerns, retailers must ensure that robust security measures are in place, including encryption and strict data protection protocols, to prevent unauthorized access and misuse of customer information [126,127,128,129].

Despite these challenges, the adoption of FacePay technology in grocery shopping is expected to grow. As consumers become more accustomed to digital and biometric payment methods, and as retailers continue to innovate and address privacy concerns, facial recognition payments may become a standard feature in grocery stores worldwide. This technology represents a significant step forward in the evolution of retail, offering a glimpse into the future of shopping where speed, convenience, and security are seamlessly integrated.

In the Table 8 there is an juxtaposition of using of facial recognition technology in the case of in-store grocery shopping.

Facial recognition technology has emerged as a transformative force across various sectors, and its application in grocery shopping is particularly noteworthy. This innovative technology offers many of benefits that enhance both the shopping experience for consumers and operational efficiencies for retailers [117,123] (Table 9). One of the most significant advantages of facial recognition technology in grocery shopping is the enhancement of customer convenience. By leveraging this technology, grocery stores can streamline the checkout process [118].

For instance, customers who opt into the facial recognition system can bypass traditional checkout lines, allowing for a seamless and swift payment experience. This not only reduces the time spent waiting in line but also alleviates congestion during peak shopping hours [119]. By facilitating quicker transactions, retailers can improve customer satisfaction, as shoppers appreciate a more efficient and hassle-free shopping experience. In addition to improving the speed and efficiency of transactions, facial recognition technology also contributes to personalized marketing efforts. By analyzing the facial features and expressions of customers, retailers can gain insights into their preferences and shopping habits. This data can be utilized to tailor marketing campaigns and promotions, ultimately leading to a more personalized shopping experience. For example, if a shopper frequently purchases organic products, the grocery store can present targeted discounts on organic items or highlight new organic arrivals through personalized advertisements. This level of customization not only enhances customer engagement but also increases the likelihood of repeat purchases [120].

Also, the implementation of facial recognition technology can significantly enhance security within grocery stores. By monitoring customer behavior and identifying potential threats, retailers can deter theft and enhance overall safety. The technology can alert staff to suspicious activities, enabling them to respond promptly and effectively. This heightened security not only protects the store’s assets but also creates a safer shopping environment for customers, fostering trust and loyalty towards the brand [121]. Another important benefit of facial recognition technology is its ability to assist in inventory management. By monitoring customer interactions with various products, retailers can gain valuable insights into which items are in high demand and which are less popular. This data can inform restocking strategies and promotional efforts, ensuring that popular items are always available while reducing waste for less popular products. Additionally, this information can assist in optimizing store layouts, as retailers can strategically place high-demand products in accessible locations, further enhancing the shopping experience [126].

Facial recognition technology can aid in fostering a sense of community within grocery shopping environments. For instance, as customers are recognized and greeted by name upon entering the store, it creates a welcoming atmosphere that enhances the overall shopping experience. This personal touch can be particularly beneficial in local grocery stores, where building relationships with customers is crucial for fostering loyalty. When shoppers feel recognized and valued, they are more likely to return, contributing to a loyal customer base [127].

The implementation of facial recognition technology in grocery stores is accompanied by a range of significant challenges that merit careful consideration (Table 10). One of the concerns pertains to privacy issues. As customers become increasingly aware of how their personal data is collected, stored, and utilized, apprehensions about surveillance and the potential misuse of their biometric information can arise [118,119]. This discomfort may lead to a reluctance among consumers to embrace such technology, ultimately impacting their shopping behaviors and brand loyalty. Also, the accuracy of facial recognition systems presents another challenge [121]. While advancements in artificial intelligence have improved the reliability of these technologies, they are not infallible. Issues such as misidentification or failure to recognize individuals, particularly in diverse populations, can lead to erroneous conclusions, causing frustration and mistrust among customers. Furthermore, technical malfunctions or inadequate lighting conditions in-stores can exacerbate these accuracy issues, further hindering the seamless integration of facial recognition technology into the shopping experience [126,127,128].

Legal and regulatory considerations pose significant challenges. As laws governing data protection and privacy continue to evolve, businesses must navigate a complex landscape to ensure compliance. The absence of standardized regulations specifically addressing facial recognition technology means that grocery retailers might face potential legal repercussions if they fail to adhere to varying regional and national guidelines. This uncertainty can discourage businesses from fully investing in such technologies, fearing the ramifications of non-compliance [129,142].

There is the ethical dimension to consider. The deployment of facial recognition technology in grocery stores raises questions about consent and the extent to which customers are informed about the use of their biometric data [121,126]. If consumers feel that they have not provided informed consent or if they perceive that their autonomy is being compromised, it could lead to public backlash and damage to a store’s reputation. Striking a balance between innovation and ethical responsibility is crucial for grocery retailers aiming to implement these advanced technologies effectively [123,127].

It should be mentioned that the operational costs associated with the deployment of facial recognition systems can be substantial. The initial investment in hardware and software, alongside ongoing maintenance and upgrades, can strain budgets, particularly for smaller grocery chains. The necessity for staff training on new systems further adds to the financial burden, creating a barrier for many retailers considering this technology [117,119,126,129].

### 3.4. Augmented Reality-Supported Shopping

The way of grocery shopping is evolving rapidly with the advent of new technologies. Among these also augmented reality (AR) stands out as a transformative innovation. This technology offers immersive and interactive shopping experiences that bridge the gap between physical stores and online shopping. AR overlays digital information on the real world, enhancing the shopping experience. Shoppers can use their smartphones or AR glasses to get additional information about products, find items in a store, or visualize products in their home environment.

Marks & Spencer (M&S) has launched a public trial of its augmented reality wayfinding app, List&Go, at its store [144]. The app allows shoppers to input a list of products and follow an on-screen path to their locations on the shelves. The app, which uses the store’s Wi-Fi network and digital planograms for navigation, is a UK first of its kind.

NISA (retail outlet in the UK) has partnered with technology company Jisp to introduce Scan&Save, an augmented reality vouchering system, in its stores across the UK [145]. This system allows customers to scan product barcodes to view promotions and redeem discount vouchers directly from their phones. The pilot run of Scan&Save saw impressive results with over 82,000 scans, 40,000 taps, and 32,000 voucher redemptions. Participating stores reported significant customer engagement, with 81% of users utilizing the system multiple times. The AR system aims to reduce carbon footprint and food waste. Retailers are compensated for each scan and redemption, incentivizing them to adopt the technology.

**Figure 4:**
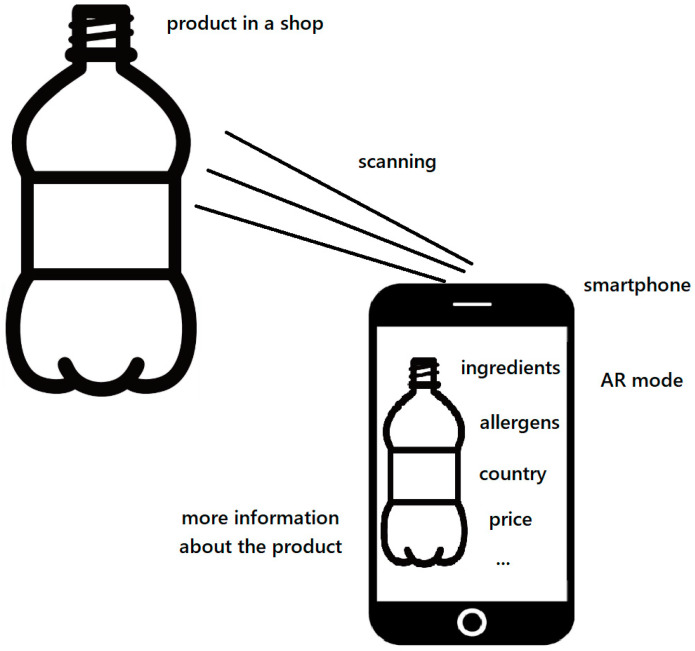
The scheme of an example use of augmented reality in a store [own work].

With limited space on product labels, brands are turning to augmented reality packaging. Customers can scan a QR code on a product label to access additional information about the item [146]. Figure 4 shows a scheme of an example use of augmented reality in a store. This is an example of scanning product for getting more information about it. The Italian sauce brand Francesco Rinaldi uses AR packaging, allowing users to scan a jar with their phones to unlock detailed information about their sauces [147]. Supermarkets can use AR packaging to provide more information about their own-brand products, such as nutritional details and sustainability practices. AR packaging also offers storytelling potential to captivate customers. For instance, Jack Daniels used WebAR to transport users to their distillery in Lynchburg, Tennessee [146].

When it comes to the scientific research that can be found in literature, Ahn et al. [148] prove that augmented reality applications on modern smartphones effectively assist grocery shoppers in making healthier decisions. Authors’ prototype AR-assisted grocery shopping app provides real-time, personalized recommendations for healthy products and flags items to avoid based on specific health concerns, such as allergies, low-sodium diets, and caloric intake. Evaluation with 15 in-person shoppers and 104 online survey participants demonstrated that the AR overlay reduces the search time for healthy items and that color-coded tags improve users’ ability to quickly identify recommended and unsuitable products. Similar research was conducted by Gutiérrez et al. [149]. The authors introduced PHARA, an augmented reality mobile assistant designed to aid in making healthier food choices at grocery stores. Using a user-centered design approach, they show that AR technology can effectively present food product information. The topic of AR fostering healthier food purchases was also presented by other authors [150,151].

In the literature some authors also show more technological approach to the topic of AR in grocery shops, presenting the innovative prototypes or practical solutions implemented. The authors of [152] present a system combining deep learning and augmented reality to enhance customer navigation and information access. The system learns the visual layout of store areas through deep learning and uses customer-uploaded images to identify their location with 98% accuracy. AR techniques then provide detailed information about the customer’s location, including routes to products, 3D product visualizations, user location, and analytics. This innovative approach aims to significantly improve the user experience in retail by offering advanced visualization, personalization, and enhanced customer interaction. In another paper [153], the authors propose a mobile application called ARGrocery that uses marker-based AR to identify products via a smartphone camera, displaying detailed information such as product name, brand, and ingredients. It also employs color-coded AR tags to help users quickly distinguish suitable from unsuitable products. This AR-enhanced system aims to enrich the shopping experience by providing comprehensive, easily accessible product information, aiding in quicker and more informed decision-making. On the other hand, there are some solutions using AR goggles instead of a mobile phone. The paper of [154] introduces CoShopper, a system combining artificial intelligence and AR to enhance grocery shopping using smart glasses. Utilizing a convolutional neural network (CNN) for item detection, the system retrieves data from extensive nutrition databases, personal medical reports, and grocery store datasets to provide real-time, user-specific nutrition facts, health tips, and warnings about unhealthy selections. CoShopper improves item detection accuracy, aids in better product selection, enhances cost efficiency, and reduces shopping time. Advani et al. [155] detail the development of the Third-Eye prototype, focusing on grocery shopping. Key features include identifying pantry items, navigating stores, locating products on shelves, and recognizing items in prepared food sections. The system employs smart glasses, a specialized glove, and a sensor-equipped shopping cart. It is mainly for the visually impaired, but not only. Ongoing improvements aim to enhance product recognition, provide dynamic recommendations, and optimize hardware for better performance.

Augmented reality applications are increasingly revolutionizing shopping experiences in supermarkets, as evidenced by diverse examples in the literature and practice (from websites of supermarkets). These solutions vary widely, showcasing AR’s versatility and potential impact. From AR wayfinding apps that guide shoppers to specific products with ease to AR vouchering systems that enhance engagement through interactive promotions, supermarkets are leveraging this technology to improve customer navigation, increase sales, and enhance overall shopping satisfaction. As these technologies continue to evolve, they are poised to redefine how consumers interact with and perceive traditional shopping environments, promising even greater convenience, personalization, and immersive shopping experiences in the future.

## 4. Discussion

The future of in-store shopping from a technological standpoint is poised to be radically different from the experience known today, driven by the continuous integration of advanced digital tools that promise to enhance convenience, personalization, and efficiency (Table 11). As technology evolves, grocery stores are expected to become increasingly smart, interconnected environments where the boundaries between the physical and digital worlds blur.

One of the most significant advancements anticipated in the near future is the widespread adoption of artificial intelligence and machine learning in various aspects of the in-store experience. AI will power sophisticated customer analytics, enabling stores to offer hyper-personalized shopping experiences. For instance, AI-driven systems could analyze a shopper’s purchasing history, dietary preferences, and even real-time behavior in the store to provide tailored recommendations, promotions, and product information directly to their smartphones or through in-store digital displays. This level of personalization will not only enhance customer satisfaction but also drive sales by ensuring that customers are presented with products that are most relevant to their needs.

Automation will also play an important role in the future of in-store shopping. Self-service technologies, such as autonomous checkout systems, are likely to become more prevalent and sophisticated. These systems will leverage AI and computer vision to allow customers to simply walk out of the store with their selected items, automatically charging their accounts without the need for a traditional checkout process. This concept, already being piloted by some leading retailers, represents a major leap toward a frictionless shopping experience.

In addition to AI and automation, augmented reality (AR) is expected to become a standard feature in many grocery stores. AR will enhance the shopping journey by overlaying digital information onto the physical environment, accessible through smartphones or wearable devices like smart glasses. Shoppers might use AR to visualize product ingredients, view interactive promotions, or navigate the store more efficiently. AR could also facilitate virtual try-ons of products, such as cosmetics or fresh produce, allowing customers to better assess the quality and suitability of items before purchasing.

The Internet of Things (IoT) will further transform in-store shopping by creating a highly interconnected retail ecosystem. Smart shelves, equipped with sensors and RFID tags, will monitor inventory levels in real time and automatically trigger restocking processes, ensuring that popular products are always available. IoT-enabled devices will also allow for dynamic pricing strategies, where prices can be adjusted in real time based on demand, expiration dates, or promotional events. This interconnected environment will provide retailers with valuable data on shopping patterns and inventory management, allowing them to optimize operations and enhance the overall customer experience.

Also, as sustainability becomes an increasingly important consideration for consumers, technology will play a key role in promoting eco-friendly shopping practices. Stores may integrate digital tools that help customers make more sustainable choices, such as carbon footprint calculators or apps that suggest products based on environmental impact. Additionally, advancements in blockchain technology could be used to provide transparent supply chain information, allowing customers to trace the origins of products and verify claims such as organic or fair trade, thus building trust and fostering more informed purchasing decisions.

The results presented within the paper can be analyzed from the TAM (technology acceptance Model) point of view, which is a well-established framework for understanding user acceptance of technology. The core components of TAM—Perceived Usefulness (PU) and Perceived Ease of Use (PEOU) [156] —play a significant role in determining how users accept and adopt new technologies, and these concepts can be applied to the technologies discussed in the paper.

Perceived Usefulness (PU) refers to the degree to which a person believes that using a particular system would enhance their performance. In the context of grocery shopping, the paper highlights several technologies that are designed to improve the shopping experience by making it more efficient and convenient [157,158,159]. For example, self-service technologies (SSTs) like self-checkouts and Scan&Go systems are presented as tools that reduce the time spent in queues and provide greater autonomy to shoppers. These features are likely to be perceived as highly useful by consumers who value time savings and the ability to manage their shopping independently. Similarly, AI-driven solutions, such as smart shopping assistants and automated checkout systems, are discussed as innovations that personalize the shopping experience and streamline the checkout process. These technologies offer tangible benefits by simplifying decision-making and improving efficiency, thereby enhancing their perceived usefulness.

Perceived Ease of Use (PEOU) is another critical factor in the TAM framework, referring to the degree to which a person believes that using a system would be free of effort [160,161]. The paper provides insights into how this factor influences the adoption of new grocery shopping technologies. For instance, while self-service technologies are designed to be efficient, their ease of use can vary significantly among different user groups. Older adults, for example, may find these systems challenging to navigate, which can negatively impact their perception of ease of use. The paper also discusses the potential difficulties associated with AI and AR/VR technologies, such as system malfunctions and the need for users to have a certain level of technological proficiency. These challenges can hinder the perceived ease of use, particularly for users who are less familiar with advanced technologies or who experience difficulties with non-intuitive interfaces.

The paper indirectly touches on consumer attitudes toward using new technologies, which TAM posits as an intermediary between perceived usefulness and ease of use and the intention to use a technology [162,163]. The mixed reactions to self-service checkout systems, for example, illustrate how consumer attitudes can vary depending on their experiences with the technology. While some users appreciate the speed and autonomy these systems offer, others may miss the human interaction typically associated with traditional checkout methods or may struggle with using the technology. These attitudes are critical in shaping users’ behavioral intentions and their eventual adoption of these technologies.

The results of the paper suggest that the adoption of technological innovations in grocery shopping is closely tied to users’ behavioral intentions, which are in turn influenced by their perceptions of usefulness and ease of use. The paper provides examples of how technologies like Scan&Go have been widely adopted in supermarkets around the world, indicating that once users perceive a technology as both useful and easy to use, their intention to continue using it increases, leading to broader acceptance and integration of the technology in everyday shopping practices.

## 5. Conclusions

The paper presents an in-depth analysis of the significant technological advancements that are transforming the traditional grocery shopping experience. The review comprehensively covers the introduction and integration of various technologies, which are increasingly being adopted by retailers to enhance customer experience and streamline shopping processes.

The paper analysis the multifaceted impact of these technologies, emphasizing their potential to revolutionize the grocery shopping landscape by making it more efficient, personalized, and engaging. Self-service technologies, such as self-checkouts and Scan&Go systems, are highlighted as pivotal innovations that empower customers by reducing wait times and providing greater autonomy during the shopping process. These systems, however, are not without their challenges, as the paper notes the varying levels of acceptance among different demographic groups, particularly older consumers who may find these technologies less intuitive or more difficult to use.

Artificial intelligence emerges as a key player in the transformation of grocery shopping, with its ability to personalize the shopping experience through smart recommendations, optimize inventory management, and automate checkout processes. The integration of AI into the retail environment offers significant benefits, including increased efficiency, reduced labor costs, and enhanced customer satisfaction. However, the paper also addresses the potential drawbacks, such as the need for robust data management and privacy concerns, which could impact consumer trust and the overall adoption of AI-driven solutions.

The exploration of augmented reality technologies within the paper adds a forward-looking dimension to the discussion. These immersive technologies have the potential to transform the way consumers interact with products, offering virtual try-ons, enhanced product information, and engaging shopping experiences that bridge the gap between online and in-store shopping. However, the paper recognizes that the widespread adoption of AR in grocery shopping is still in its nascent stages, with significant hurdles to overcome, including the cost of implementation, technological limitations, and the need for consumer education to ensure these technologies are both accessible and intuitive.

A recurring theme throughout the paper is the critical importance of consumer acceptance in the successful implementation of these technologies. The discussion implicitly aligns with the principles of the Technology Acceptance Model (TAM), which posits that perceived usefulness and perceived ease of use are key determinants of technology adoption. The paper provides numerous examples illustrating how these factors play out in the context of grocery shopping technologies. For instance, while SSTs and AI-driven solutions may offer substantial benefits, their ultimate success depends on how consumers perceive their value and ease of use. Technologies that are perceived as difficult to use or that fail to clearly demonstrate their utility are less likely to be embraced by consumers, regardless of their potential advantages.

Also, the paper highlights the broader implications of digital transformation in grocery shopping, suggesting that these technological innovations could lead to a fundamental shift in consumer behavior and expectations. As retailers continue to adopt and refine these technologies, they must consider not only the operational efficiencies they provide but also the impact on customer experience and satisfaction. The paper suggests that a balanced approach, which combines the benefits of technological innovation with a deep understanding of consumer needs and preferences, will be essential for the successful integration of these new tools into the grocery shopping environment.

The scientific contribution of the paper is twofold: first, it offers a detailed review of current technologies, identifying both their benefits and limitations in the context of grocery shopping; second, it provides a framework for understanding the factors that influence the adoption and success of these technologies, implicitly drawing on theories such as the Technology Acceptance Model.

Through its analysis, the paper highlights the dynamic interplay between technological innovation and consumer acceptance, emphasizing that the success of these new tools depends not only on their technical capabilities but also on how they are perceived and used by consumers. This perspective is scientifically valuable as it bridges the gap between technological advancements and consumer behavior, offering insights that are critical for the successful integration of these technologies into the retail environment. Additionally, the paper’s forward-looking approach, particularly its discussion of emerging technologies like AR contributes to the ongoing discourse on the future of retail, providing a foundation for further research in this rapidly evolving field. Overall, the scientific value of this paper is its ability to offer a nuanced understanding of the digital transformation in grocery shopping, with implications for both academic research and practical applications in the retail industry.

The paper’s theoretical contribution is its ability to contextualize the adoption of advanced grocery shopping technologies within established theoretical frameworks, while also pushing the boundaries of these theories to accommodate the unique challenges and opportunities presented by digital transformation in the retail sector. This integration of theory and practice provides a foundation for future research and offers valuable insights into the factors that drive or hinder the successful adoption of new technologies in consumer markets.

## Figures and Tables

**Table 1:** Examples of supermarkets that offers Scan&Go technology for grocery shopping [own work].

Name of Supermarket/Shop	Name of Technology/Application	Example of Country	Reference	Website with Description of Technology
7-Eleven	Mobile Checkout/Just Scan, Pay & Go	USA	[16]	https://www.7-eleven.com/7rewards/mobile-checkout (accessed on 18 August 2024.)
ALDI	Shop&GO	UK	[17]	https://groceries.aldi.co.uk/en-GB/shopandgo (accessed on 18 August 2024.)
ASDA	Scan&Go	UK	[18]	https://www.asda.com/about/instore/scan-and-go (accessed on 18 August 2024.)
Auchan	Scan to Go	France	[19]	https://www.auchan.fr/scan-to-go/ep-scan-to-go (accessed on 18 August 2024.)
Carrefour	Scan&Go	Poland	[20]	https://www.carrefour.pl/lp-scan-go-kupuj-szybko-i-wygodnie (accessed on 18 August 2024.)
Coop	Scan&Pay	Sweden	[21]	https://www.coop.se/medlem/tjanster--verktyg/scan--pay/ (accessed on 18 August 2024.)
Kaufland	K-Scan	Germany	[22]	https://www.kaufland.pl/dla-klienta/nasze-uslugi/k-scan.html (accessed on 18 August 2024.)
Sainsbury’s	Scan & bag as you Go	UK	[23]	https://smartshop.sainsburys.co.uk/ (accessed on 18 August 2024.)
Walmart	Scan&Go	USA	[24]	https://www.walmart.com/cp/scan-go/9679980 (accessed on 18 August 2024.)

**Table 2:** Examples of AI usage-in-store grocery shopping.

The Use of AI	Description
Smart Shopping Assistants	AI-powered shopping assistants can be deployed in-store through kiosks or mobile applications, helping customers locate products, compare prices, and provide personalized recommendations based on their shopping history and preferences. This enhances the overall shopping experience by making it more interactive and efficient.
Inventory Management and Stock Alerts	AI algorithms can analyze inventory levels and predict demand for various products, ensuring that shelves are stocked appropriately. During the shopping process, customers can receive real-time alerts on product availability and suggested alternatives if items are out of stock, helping to streamline their buying decisions.
Dynamic Pricing Strategies	AI can analyze market trends, competitor prices, and customer behavior to adjust prices dynamically. This allows grocery stores to offer competitive pricing during peak shopping hours or for specific customer segments, encouraging purchases during the buying process.
Automated Checkout Solutions	AI-driven checkout systems can utilize image recognition technology to scan items as customers place them in their carts. This eliminates the need for manual scanning at self-checkout kiosks, speeding up the checkout process and reducing queues.
Personalized Shopping Experience	By leveraging customer data and shopping habits, AI can provide tailored product suggestions and personalized promotions in real-time. This enhances customer engagement and increases the likelihood of additional purchases during the shopping process.
In-Store Navigation Apps	AI-powered navigation apps can guide customers through the store based on their shopping lists, highlighting the quickest routes to desired products. This can significantly enhance efficiency, especially in larger grocery stores.
Customer Sentiment Analysis	AI can analyze customer feedback and sentiment through various channels, helping retailers understand shopper preferences and pain points. This information can be used to adjust in-store layouts, product offerings, and marketing strategies to enhance the overall buying experience.
Fraud Detection and Prevention	AI systems can monitor transactions and customer behavior to identify patterns indicative of fraudulent activities, ensuring security during the buying process. This helps protect both retailers and customers from potential fraud.
Automated Stock Replenishment	AI can predict when certain products will run low based on sales data and seasonal trends, automatically triggering replenishment orders. This ensures that popular items are always available for customers during their shopping trips.

Source: Author’s own work on basis [9,25,26,27,28,29,30,31,32,33,34,35,36,37,38,39,40,41,42,43,44,45,46,47,48,49,50,51,52,53,54,55,56].

**Table 3:** Examples of using smart shopping-in-store grocery shopping.

Name of Supermarket/Shop	Name of Technology/Application	Example of Country	Description	References
Amazon Fresh	Amazon Alexa Integration	USA	Amazon Fresh locations allow customers to use Alexa to ask for product locations, receive recipe suggestions, and create shopping lists. This voice-activated technology provides a hands-free shopping experience, enhancing convenience and engagement in-store.	[57,58]
Walmart	Walmart Mobile App	USA	The Walmart app includes a shopping assistant feature that helps customers create shopping lists and guides them through the store using location-based services. It displays the fastest routes to find items, improving efficiency and reducing shopping time.	[59,60]
Kroger	Kroger Mobile App	USA	The Kroger app features a smart shopping assistant that allows customers to access product information, check prices, and find promotions. The app uses personalized recommendations to enhance the shopping experience and encourage discovery of new products.	[61,62]
Albertsons	Albertsons Mobile App	USA	The Albertsons app provides a shopping assistant that enables customers to scan items for prices and nutritional information. It also offers personalized deals and suggestions based on previous purchases, fostering a more tailored shopping experience.	[63,64]
Tesco	Tesco Clubcard App	UK	The Tesco Clubcard app functions as a smart shopping assistant by providing personalized discounts and recommendations based on customer purchase history. It also allows customers to create shopping lists and access store maps for easier navigation.	[65,66]
Sainsbury’s	Sainsbury’s Smart Shop	UK	Sainsbury’s offers a Smart Shop feature in its mobile app, allowing customers to scan items as they shop, check prices, and receive personalized offers. This technology enhances the shopping process by streamlining transactions and providing real-time information.	[67,68]
Lidl	Lidl Plus App	Various Countries	The Lidl Plus app serves as a shopping assistant by offering digital coupons, personalized offers, and product information. Customers can create shopping lists within the app, which helps them save money and find items efficiently while shopping.	[69,70]
Ahold Delhaize	Peapod Mobile App	USA	Ahold Delhaize’s Peapod app functions as a smart shopping assistant by enabling customers to order groceries online and schedule in-store pickups. It provides product suggestions based on previous purchases, making the grocery shopping process more convenient.	[71,72]

Source: Author’s own work on basis [57,58,59,60,61,62,63,64,65,66,67,68,69,70,71,72].

**Table 4:** Examples of using dynamic pricing strategy-in-store grocery shopping.

Name of Supermarket/Shop	Example of Country	Description	References
Carrefour	Poland	Carrefour utilizes digital price tags that enable real-time price updates across its stores. This allows the retailer to adjust prices for near-expiration products or respond to market conditions quickly, helping to reduce waste and attract price-sensitive customers.	[73,74]
Żabka	Poland	Zabka employs data analytics to adjust prices based on foot traffic and sales trends. By offering discounts during off-peak hours, Zabka optimizes sales and enhances customer traffic, making shopping more appealing during quieter times.	[75]
Walmart	USA	Walmart uses advanced algorithms to monitor competitor prices and automatically adjust its pricing in real-time. This strategy allows Walmart to remain competitive while maximizing profit margins, ensuring customers perceive it as a cost-effective shopping option.	[76]
Tesco	UK	Tesco leverages its Clubcard loyalty program to analyze customer purchase histories and tailor discounts. This personalized approach encourages customer loyalty by offering targeted promotions based on individual shopping habits.	[77]
Aldi	Germany	Aldi implements a flexible pricing strategy by analyzing competitor pricing and demand fluctuations. The retailer may adjust prices on popular items based on local market conditions, ensuring competitive pricing while maintaining profitability.	[78,79]
Lidl	Various Countries	Lidl utilizes a dynamic pricing model that allows real-time adjustments based on stock levels and customer demand. This approach helps manage inventory effectively while also responding to changing customer preferences.	[80,81]

Source: Author’s own work on basis [73,74,75,76,77,78,79,80,81].

**Table 5:** Examples of using AiFi technology-in-store grocery shopping.

Name of Supermarket/Shop	Example of Country	Description	References
Zabka	Poland	Zabka has begun testing cashier-less stores that utilize AiFi technology to provide a seamless shopping experience. Customers can enter the store, select items, and leave without going through a traditional checkout, as the system automatically tracks their purchases.	[93,94]
Amazon Go	USA	Amazon Go stores employ AiFi-like technology to enable customers to shop and leave without checking out. Cameras and sensors detect the items customers take, and the total is charged to their Amazon account automatically when they exit the store.	[95,96]
Carrefour	France	Carrefour is experimenting with automated stores featuring AiFi technology, allowing customers to scan items as they shop and automatically calculate their total. This technology enhances the shopping experience by eliminating traditional checkout lines.	[97,98]
Ahold Delhaize	The Netherlands	Ahold Delhaize is exploring automated stores that use AiFi technology to track purchases in real-time. Shoppers can select items, and their accounts are charged automatically as they leave, streamlining the shopping process.	[99,100]
Sainsbury’s	UK	Sainsbury’s is testing AiFi technology in select locations, enabling customers to shop without traditional checkouts. The system monitors purchases automatically and charges customers as they exit, improving the overall shopping experience.	[101,102]
7-Eleven	Japan	7-Eleven has implemented AiFi technology in select locations, allowing customers to shop and leave without going through a cashier. The system automatically detects items and processes payments via mobile apps, enhancing convenience for shoppers.	[103,104]
Spar	Austria	Spar has begun using AiFi technology in some locations to create a cashier-less shopping environment. Customers can shop freely, and the system tracks purchases, charging their accounts automatically as they exit the store.	[105]
Lidl	Germany	Lidl is experimenting with automated checkout solutions that use AiFi technology in select locations, enabling customers to scan items as they shop and facilitating a quick exit without traditional checkout processes.	[106]

Source: Author’s own work on basis [93,94,95,96,97,98,99,100,101,102,103,104,105,106].

**Table 6:** Benefits of using AI technology-in-store grocery shopping.

Benefit	Description
Enhanced Customer Experience	AI technology can personalize the shopping experience by analyzing customer preferences and behaviors. This allows for tailored product recommendations, targeted promotions, and improved in-store navigation, ultimately leading to increased customer satisfaction and loyalty.
Improved Inventory Management	AI algorithms can analyze sales data, seasonal trends, and customer demand to optimize inventory levels. This helps grocery stores maintain adequate stock, reduce waste from expired items, and streamline supply chain operations, ensuring that popular products are always available for customers.
Dynamic Pricing Strategies	AI can enable dynamic pricing by analyzing market trends, competitor pricing, and customer behavior in real-time. This allows grocery stores to adjust prices based on demand, optimize sales, and maximize profit margins while providing competitive pricing to customers.
Efficient Checkout Processes	AI-driven systems can streamline checkout processes through automated checkouts and facial recognition technology. This reduces wait times for customers, enhances operational efficiency, and improves the overall shopping experience by allowing faster transactions.
Predictive Analytics for Sales	AI can leverage predictive analytics to forecast future sales trends based on historical data, customer behavior, and external factors. This insight enables grocery stores to make informed decisions about promotions, inventory levels, and marketing strategies, ultimately driving sales growth.
Enhanced Security and Fraud Detection	AI technology can improve store security by analyzing video footage for suspicious activity or unusual patterns in customer behavior. This helps prevent theft and fraud, ensuring a safer shopping environment for both customers and employees.
Personalized Marketing Campaigns	AI can analyze customer purchase history and preferences to develop targeted marketing campaigns that resonate with specific customer segments. This personalized approach can increase engagement and conversion rates, driving more sales and fostering brand loyalty.
Streamlined Supply Chain Management	AI can optimize supply chain logistics by predicting demand fluctuations and analyzing transportation routes. This leads to improved efficiency in stock replenishment and reduced operational costs, ensuring that stores are stocked with the right products at the right time.

Source: Author’s own work on basis [9,25,26,27,28,29,30,31,32,33,34,35,36,37,38,39,40,41,42,43,44,46,47,48,49,50,51,52,53,54,55,82,83,85,86,87,88,89,90,91,92,106,107,108,109,110].

**Table 8:** Examples of using facial recognition technology-in-store grocery shopping.

Name of Supermarket/Shop	Example of Country	Description	Reference
Empik	Poland	Empik has implemented FacePay technology in some stores, allowing customers to pay using facial recognition. This modern approach enhances the convenience and speed of transactions, particularly in high-traffic areas.	[130]
Hema Supermarkets (Alibaba)	China	Operated by Alibaba, Hema Supermarkets use Alipay’s facial recognition for seamless payments. The system is part of an integrated high-tech shopping experience, which includes features like automated checkout and personalized recommendations.	[131]
Auchan Retail	France, Poland, other European countries	Auchan is testing facial recognition payments across its stores as part of its digital retail strategy. This initiative aims to reduce checkout times and offer a more efficient, contactless shopping experience.	[122]
Metro AG	Germany	Metro AG has piloted facial recognition payments in some of its cash-and-carry stores. This technology is designed to streamline the checkout process and improve the overall customer experience by using biometrics for secure transactions.	[124]
7-Eleven	South Korea Thailand Japan	7-Eleven’s unmanned stores in South Korea use facial recognition technology for quick and easy payments, particularly in urban areas where convenience is key. The system allows for a completely cashier-less shopping experience.	[132,133,134]
KFC (Yum China)	China	KFC in China has partnered with Alipay to introduce the “Smile to Pay” system, where customers can pay for their orders using facial recognition technology by simply smiling at a camera. This method is fast, secure, and reduces physical contact.	[135,136]
Lotte Mart	South Korea	Lotte Mart, a major retail chain in South Korea, has introduced facial recognition payment systems in some of its stores. The system is integrated with customer loyalty programs and provides a swift, contactless checkout process.	[137]
Carrefour	UAE	Carrefour in China has adopted Alipay’s facial recognition technology for payments in some stores. This system is part of Carrefour’s strategy to enhance the shopping experience through digital innovation and provide a fast, convenient way for customers to pay.	[138,139]
SPAR	UK	SPAR in Russia has implemented facial recognition technology in select stores. This system is linked to customer accounts and allows for quick, secure payments by scanning the customer’s face at checkout.	[140,141]

Source: Author’s own work on basis [122,124,130,131,132,133,134,135,136,137,138,139,140,141].

**Table 9:** Benefits of using facial recognition technology in grocery shopping.

Benefit	Description
Increased Convenience	Facial recognition technology allows customers to complete transactions quickly without needing cash, cards, or mobile devices. This streamlines the checkout process, reducing wait times and improving the overall shopping experience.
Enhanced Security	Facial recognition provides a secure method of payment by verifying a customer’s identity through unique biometric data, reducing the risk of fraud and unauthorized transactions.
Contactless Payment	The technology enables a fully contactless payment experience, which is particularly valuable in maintaining hygiene and safety standards, especially in the context of health concerns such as the COVID-19 pandemic.
Personalization	Facial recognition can be integrated with customer profiles, allowing for personalized shopping experiences, such as tailored promotions, loyalty rewards, and product recommendations based on past purchases.
Faster Checkout	By automating the payment process, facial recognition technology significantly speeds up the checkout process, reducing lines and enhancing customer satisfaction.
Integration with Loyalty Programs	The technology can be linked to customer loyalty programs, automatically applying discounts and tracking purchases without requiring physical cards or manual input.
Reduced Operational Costs	For retailers, facial recognition can help reduce operational costs by minimizing the need for cashiers and reducing the likelihood of human error during transactions.

Source: Author’s own work on basis [114,117,118,119,120,121,123,126,127,128,129,142,143].

**Table 10:** Challenges of using facial recognition technology in grocery shopping.

Challenge	Description	Methods of Overcoming
Privacy Concerns	Collecting and storing facial biometric data raises significant privacy issues, as customers may be concerned about how their data is used and who has access to it.	Implementing strict data protection policies, using encryption, and offering transparency in data usage. Customers should also have the option to opt-in or opt-out of the service.
Data Security Risks	Facial recognition systems are vulnerable to cyberattacks, where sensitive biometric data could be stolen or misused.	Utilizing advanced cybersecurity measures, including encryption, regular security audits, and multi-factor authentication to safeguard biometric data.
Consumer Trust and Acceptance	Consumers may be hesitant to use facial recognition technology due to concerns about accuracy, misuse, or unfamiliarity with the technology.	Educating customers about the technology’s benefits, ensuring high accuracy rates, and providing clear information on how the system works, along with offering alternatives to those uncomfortable with the technology.
Regulatory Compliance	Different countries and regions have varying laws regarding the use of biometric data, making it challenging to ensure compliance across all locations.	Adhering to local data protection laws, conducting regular legal reviews, and ensuring that the technology is implemented in a way that complies with regional regulations.
Technical Limitations	The accuracy of facial recognition can be affected by factors like lighting conditions, changes in appearance, and the quality of the camera system.	Investing in high-quality camera systems, using AI to improve recognition accuracy, and incorporating fallback options like manual verification when needed.
Implementation Costs	The initial setup and ongoing maintenance of facial recognition systems can be expensive for retailers, particularly smaller businesses.	Seeking cost-effective solutions, considering phased rollouts, and evaluating the return on investment through improved customer

Source: Author’s own work on basis [114,117,118,119,120,121,123,126,127,128,129,142,143].

**Table 11:** Future trends-in-store grocery shopping.

Trend	Description
Artificial Intelligence integration	AI will be used to personalize the shopping experience by offering real-time product recommendations, optimizing inventory, and enhancing customer service through smart assistants.
Autonomous checkout Systems	The implementation of checkout-free technology where customers can automatically be charged for items as they exit the store, eliminating traditional checkouts.
Augmented reality	AR will provide an interactive shopping experience by overlaying digital information, such as product details, reviews, and personalized offers, directly onto physical products in the store.
Smart shelves and Inventory management	Shelves equipped with sensors and RFID technology will monitor inventory in real time, automatically reorder stock, and display dynamic pricing or promotions to customers.
Enhanced customer analytics	Advanced data analytics will allow stores to track and analyze customer behavior in real time, enabling more effective product placement, promotions, and personalized marketing.
Contactless and biometric Payments	The expansion of contactless payment methods, including mobile wallets and biometric authentication (e.g., facial recognition or fingerprint scanning), to provide a faster, more secure checkout experience.
Sustainability and eco-friendly technologies	Technologies like blockchain and digital product information will allow consumers to trace the sustainability of products, encouraging eco-conscious purchasing decisions.
In-store navigation and guidance systems	AI-powered navigation tools and apps will guide customers through the store, suggesting optimal shopping routes and helping them find products quickly.
Interactive digital displays	Large digital displays in stores will provide customers with real-time information, personalized offers, and interactive content that enhances the shopping experience.
Robotic assistants	In-store robots will assist with tasks such as stocking shelves, guiding customers to products, and providing information or assistance, enhancing operational efficiency.

Source: Author’s own work on basis [27,28,29,39,40,41,42,43,51,82,83,84,85,86].
